# Comparison of Clinical Manifestations and Outcomes between Hepatitis B Virus- and Hepatitis C Virus-Related Hepatocellular Carcinoma: Analysis of a Nationwide Cohort

**DOI:** 10.1371/journal.pone.0112184

**Published:** 2014-11-05

**Authors:** Dong Hyun Sinn, Geum-Youn Gwak, Juhee Cho, Seung Woon Paik, Byung Chul Yoo

**Affiliations:** 1 Department of Medicine, Samsung Medical Center, Sungkyunkwan University School of Medicine, Seoul, Korea; 2 Department of Health Science and Technology, Samsung Advanced Institute for Health Science and Technology, Sungkyunkwan University, Seoul, Korea; 3 Department of Health, Behavior and Society and Epidemiology, Johns Hopkins Bloomberg School of Public Health, Baltimore, United States of America; CEA, France

## Abstract

**Background:**

We analyzed whether difference exist in the clinical manifestations and outcomes of hepatocellular carcinoma (HCC) according to the two major etiologies of HCC from a nationwide, population-based, random HCC registry.

**Methods:**

Of the 31,521 new HCC cases registered at the Korea Central Cancer Registry between 2003 and 2005, 4,630 (14.7%) were randomly abstracted, and followed up until December 2011. Of those, 2,785 hepatitis B virus (HBV)-related and 447 hepatitis C virus (HCV)-related HCC patients were compared.

**Results:**

The mean annual incidence rates of HBV- and HCV-related HCC incidence per 100,000 persons were 20.8 and 4.9, respectively. The annual incidence rate of HBV-related HCC peaked at 50–59 age group (46.5 per 100,000 persons), while the annual incidence rate of HCV-related HCC increased gradually to the ≥70 year age group (13.2 per 100,000 persons). Large tumors (≥5 cm) and portal vein invasion at initial diagnosis were more frequent in HBV-related HCC, while multiple tumors were more frequent in HCV-related HCC. In outcome analysis, HBV-related HCC showed poorer survival than HCV-related HCC [median survival: 1.34 vs. 2.17 years, adjusted hazard ratio (95% confidence interval): 0.88 (0.78–0.98), *P* = 0.03, adjusted for age, gender, Child-Pugh class, AJCC/mUICC stage, and initial treatment modality]. However, when divided according to the AJCC/mUICC stage, survival difference was observed only for those with AJCC/mUICC stage IV tumor, but not for AJCC/mUICC stage I, II or III tumors. The treatment outcome of each modality (resection, ablation, and transartherial chemoeombolization) was comparable between the two etiologies.

**Conclusion:**

HBV-related and HCV-related HCC have clear differences in clinical manifestation, requiring different screening strategy according to etiology to optimize HCC surveillance in HBV-endemic area. However, etiology did not affect treatment outcomes and long-term survival within the same stage except for far advanced tumors.

## Introduction

Hepatocellular carcinoma (HCC) is the fifth most common cancer and the third leading cause of cancer-related deaths in the world [1]. HCC continues to increase progressively in incidence, being a major global health problem [2], [3]. The two most important risk factors for HCC are the hepatitis B virus (HBV) and hepatitis C virus (HCV) infection [4]. Worldwide, approximately 54% of cases are attributed to HBV infection (which affects 400 million people globally), while 31% are attributed to HCV infection (170 million) [5]. HCC is a major cancer in Korea [6], with a high incidence rate [2]. High incidence rate of HCC closely reflects a high prevalence rate of HBV or HCV infection [2]. Korea is an endemic area of HBV infection [7], although the prevalence of HBV is rapidly decreasing due to a nationwide universal vaccination program [8]. HBV accounts for 68∼78% of all HCC diagnosed in Korea [9]. Almost all Korean chronic hepatitis B patients are infected with HBV genotype C [10], which progresses more rapidly to HCC [8]. HCV accounts for 10∼15% of HCC diagnosed in Korea [11], and >95% of Korean chronic hepatitis C patients are infected with HCV genotype 1b or 2 [12].

Despite the histological similarities in the resulting carcinomas, there are evidences suggesting two distinct oncogenic pathways and natural histories between these major etiologies of HCC. In HBV infection, in addition to chronic inflammation, the integration of HBV DNA into the host hepatocyte DNA and the expression of viral proteins, which transactivate human oncogenes, may play a role in hepatocarcinogenesis, while in HCV infection, chronic inflammation seems to play a main role in oncogenesis [13]–[15]. Thus, cirrhosis almost always accompanies HCV-related HCC [16], but not in HBV-related HCC [17], [18]. HBV infection usually occurs in the perinatal period in an endemic area, while the immune status of the host is still immature [8]. Meanwhile, HCV infection occurs in adults with a fully matured immune system [19]. A combination of virus-specific, host genetic, environmental, and immune-related factors will affect the HCC manifestations, thus these differences in hepatocarcinogenesis may affect clinical manifestations as well as patients outcome. Indeed, several previous studies have assessed the impact of viral etiology on clinical manifestation and long-term outcome [20]–[22]. However, still controversies exist whether different HCC surveillance and management strategies according to the viral etiologies are needed. This question has not been answered, in part, because most studies were performed on a single hospital base which inevitably has a selection bias, with limited sample size and limited follow-up period. The sample sizes were 205, 359 and 127 in reports by Shiratori et al.'s [20], Tanabe et al.'s [21], and Hiotis et al.'s [22], respectively. Therefore, in this study, we used data from population-based nationwide cancer registry which has less selection bias, with large study sample and long-term follow-up period, and assessed whether true differences exist in clinical manifestations and long-term outcomes of HCC patients between the two viral etiologies.

## Methods

### Data source

In Korea, the largest government-endorsed, population-based cancer registry, called the Korea Central Cancer Registry (KCCR), was established in 1980. Patients diagnosed with cancer receive additional economic assistance from the National Health Insurance Service when registered at the KCCR, hence, almost all incidences of cancer (>95%) occurring in the population are included in the registry [23]. Therefore, the KCCR has the advantage that it has high case completeness as a cancer registry. However, the KCCR do not collect detailed information, such as liver function, tumor characteristics, treatment modalities, etc. To fully collect detailed clinical, tumor characteristics as well as treatment information and long-term outcomes of Korean HCC patients, the Korean Liver Cancer Study Group (KLCSG) built a randomly selected, population based HCC cohort based on KCCR registry (KLCSG random cohort).

The KLCSG random cohort was built as follows: Between 2003 and 2005, a total of 31,521 new cases of HCC were registered at the KCCR from about 500 hospitals nationwide. First, 25–30 hospitals were randomly selected after being stratified by region and number of cases registered, then 16.5% of HCC cases from each year were randomly selected, this gave a total of 5,262 HCC cases. Three trained abstractors visited 32 hospitals throughout the country between May 2009 and May 2010, and obtained information of each case regarding the clinical and tumor characteristics and treatment modality. Among the 5,262 cases, abstraction was possible for 4,630 HCC cases (14.7%). The major reasons of abstraction failure included malignancy other than HCC (e.g., intrahepatic cholangiocarcinoma, hepatoblastoma, etc…), data duplication or unavailable data, etc. After excluding 110 cases, which the date of the diagnosis was not between 2003 and 2005, a total of 4,520 patients were enrolled in the KLCSG random cohort. The diagnosis of HCC was made clinically or histologically based on the guidelines proposed by the KLCSG and the National Cancer Center [6]. In brief, clinical diagnosis of HCC was defined by one imaging technique (spiral CT scan, dynamic MRI, or hepatic artery angiography) showing a compatible feature of HCC in patients with alpha-fetoprotein level of more than 400 ng/ml or two imaging techniques showing compatible feature of HCC in patients with alpha-fetoprotein level of less than 400 ng/ml [6].

Out of the 4,520 patients, we analyzed 3,232 patients with either HBV or HCV infection (HBV: 2,785, HCV: 447). HBV-related HCC was defined when the presence of hepatitis B surface antigen was documented with a history of chronic liver disease. HCV-related HCC was defined when the presence of HCV RNA or anti-HCV were documented with a history of chronic liver disease. Forty-one patients had dual HBV and HCV infection, and were excluded from the analysis. The study protocol was reviewed and approved by the Institutional Review Board at Samsung Medical Center. The requirement for the informed consent was exempted by the Institutional Review Board because the study was based on the retrospective analysis of existing administrative and clinical data. Patient records/information was anonymized and de-identified prior to analysis

### Variables

The KLCSG random cohort registry collected information including age, gender, date of diagnosis, etiology, Child-Pugh class, tumor number, tumor size, presence of portal vein invasion and extrahepatic spread, American Joint Committee on Cancer (AJCC)/International Union Against Cancer (UICC) tumor-node-metastasis (TNM) stage, and applied treatment modality. At the time of data abstraction, Barcelona Clinic Liver Cancer (BCLC) staging and performance status was not collected. Hence, we re-coded the BCLC stage with Child-Pugh class, tumor size, tumor number and presence of portal vein invasion and extrahepatic spread, omitting performance status. Surgical resection, local ablation and transplantation were defined as curative therapies; and transarterial chemoembolization (TACE), transarterial chemoinfusion (TACI), systemic chemotherapy and radiation were regarded as palliative therapies. Survival data was collected from the National Statistics Service, and survival data of each patient were acquired up to December 2011.

### Statistical analysis

Statistical analysis was performed using the chi-square test to compare discrete variables and the t-test for continuous variables between HBV and HCV-related HCC. The survival rate was calculated and plotted by using the Kaplan-Meier method. To assess whether etiology was associated with survival, the Cox proportional hazard model was used, and a *P* value of less than 0.05 was considered to be significant.

## Results

### Clinical characteristics

The baseline characteristics at diagnosis are compared in Table 1. Mean age at diagnosis was much younger in HBV than HCV-related HCC (53.9±9.7 vs. 65.4±8.8, *P*<0.01). In HBV-related HCC, a comparably significant proportion of patients were under 40 years old (7%). Sixty-five percent were age between 40 and 59, and 28% were over 60 years. While in HCV-related HCC, those diagnosed below the age of 40 years was rare (1%) and most patients (77%) were over 60 years. In HBV-related HCC, the mean age at diagnosis was much younger for men than women (53.2±9.3 vs. 57.2±10.7, *P*<0.01), while in HCV-related HCC, the mean age at diagnosis was marginally younger for men than women (64.9±8.6 vs. 66.9±9.4 for men vs. women, *P* = 0.05).

**Table 1:** Characteristics of study population.

Characteristics	HBV	HCV	*P*-value
Number	2,785	447	
Age (years, mean ± S.D)	53.9±9.7	65.4±8.8	<0.01
<40	193 (7%)	4 (1%)	
40–49	800 (29%)	22 (5%)	
50–59	1,010 (36%)	77 (17%)	
60–69	647 (23%)	197 (44%)	
≥70	135 (5%)	147 (33%)	
Male, n (%)	2,248 (81%)	339 (76%)	0.02
Laboratory values			
Albumin (g/dl)	3.5 (3.0–3.9)	3.4 (3.0–3.8)	<0.01
Total bilirubin (mg/dl)	1.1 (0.7–1.7)	1.0 (0.7–1.6)	0.33
Prothrombin time (INR)	1.15 (1.05–1.29)	1.15 (1.05–1.28)	0.67
Child-Pugh class			0.18
A	1,798 (65%)	299 (67%)	
B	734 (26%)	119 (27%)	
C	254 (9%)	29 (7%)	
Diagnostic methods			0.86
Histologic	458 (16%)	75 (17%)	
Clinical	2,327 (84%)	372 (83%)	
Tumor number			0.03
Single	1,972 (71%)	294 (66%)	
Multiple	813 (29%)	153 (34%)	
Maximal tumor size (cm)			<0.01
<2	387 (14%)	79 (18%)	
2–5	1,089 (39%)	217 (49%)	
≥5	1,309 (47%)	151 (34%)	
Portal vein invasion			<0.01
Yes	739 (27%)	73 (16%)	
Extrahepatic spread	316 (11%)	40 (9%)	0.13
AJCC/mUICC stage			0.08
I	294 (11%)	54 (12%)	
II	1,171 (42%)	206 (46%)	
III	776 (28%)	120 (27%)	
IV-A	299 (11%)	43 (10%)	
IV-B	245 (9%)	24 (5%)	
BCLC stage†			<0.01
O	202 (7%)	39 (9%)	
A	1,266 (46%)	233 (52%)	
B	255 (9%)	62 (14%)	
C	808 (29%)	84 (19%)	
D	254 (9%)	29 (7%)	
Milan criteria			
Within Milan (YES)	1,250 (45%)	239 (54%)	0.01
Treatment			0.53
Curative	516 (19%)	92 (21%)	
Palliative	1,597 (57%)	246 (55%)	
Experimental*	2 (0.1%)	1 (0.2%)	
None	670 (24%)	108 (24%)	
Specific modality			0.01
Resection	295 (14%)	34 (10%)	
Ablation	194 (9%)	54 (16%)	
Transplantation	27 (1%)	4 (1%)	
TACE	1,439 (68%)	231 (68%)	
TACI	100 (5%)	13 (4%)	
Systemic chemotherapy	30 (1%)	1 (0.3%)	
Radiation	28 (1%)	1 (0.3%)	

Abbreviation: HBV, hepatitis B virus; HCV, hepatitis C virus; S.D, standard deviation; INR, international normalized ratio; AJCC/mUICC, American Joint Committee on Cancer/International Union Against Cancer; BCLC, Barcelona Clinic Liver Cancer; TACE, transarterial chemoembolization; TACI, transarterial chemoinfusion.

*These 3 patients received ^166^holmium injection therapy. Values are expressed as mean ± standard deviation, median (quartile), or no (%).

† BCLC stage and performance status was not collected at the time of data collection. Hence, BCLC stage was re-coded (staged) by authors with Child-Pugh class, tumor size, tumor number and presence of portal vein invasion and extrahepatic spread, without performance status.

**Figure 1:**
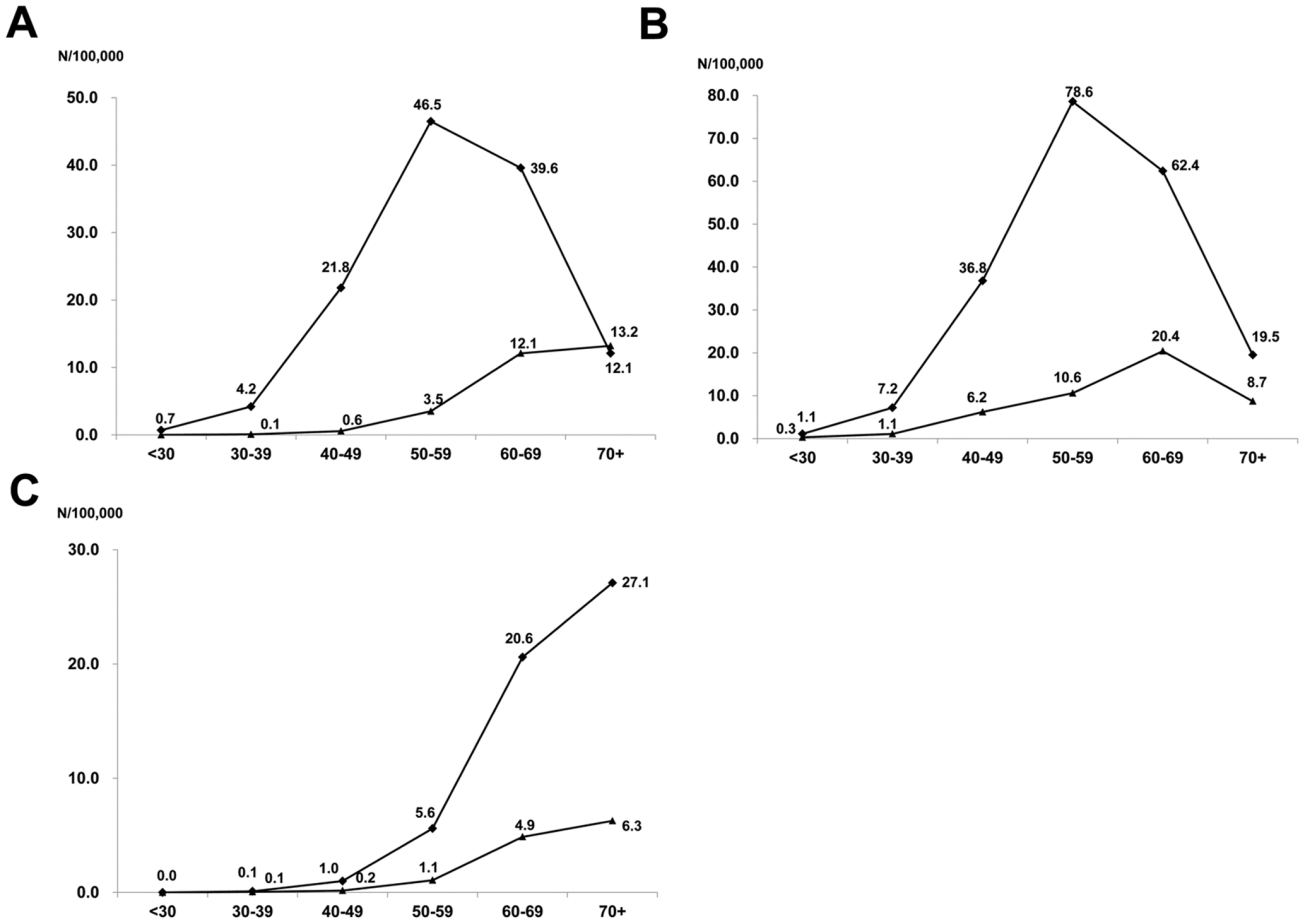
The age-specific incidence rates of hepatocellular carcinoma (HCC) by the etiology (A), by gender in HBV-related HCC (B) and by gender in HCV-related HCC (C). The annual incidence rates of HBV-related HCC peaked in the 50–59 age group, while the annual incidence rates of HCV-related HCC kept gradually increasing until age ≥70 s. Similar trend was observed after stratified by gender, although the peak mean annual incidence rates was observed in the 50–59 in men and in the 60–69 in women in HBV-related HCC (B). Diamonds (♦) and triangles (▴) represent for HBV and HCV-related HCC in (A), men and women in (B) and (C), respectively.

We estimated the annual incidence rate of HCC using the mid-point-population of the study period (data not shown, available at National statistics service, http://kostat.go.kr). The mean annual incidence rates of HBV- and HCV-related HCC per 100,000 persons were 20.8 and 4.9, respectively. The mean annual incidence rates of men per 100,000 were 34.3 and 9.1 for HBV- and HCV-related HCC, respectively, and were 7.9 and 2.1 for HBV- and HCV-related HCC for women. The annual incidence rate of HBV-related HCC peaked at age group 50–59, while the annual incidence rates of HCV-related HCC gradually increased until age group over 70 (Fig. 1A). In HBV-related HCC, the annual incidence rate peaked at 50–59 (78.6 per 100,000) in men, while it was 60–69 (20.4 per 100,000) in women (Fig. 1B). In HCV-related HCC, the annual incidence rate gradually increased until age over 70 in both genders (Fig. 1C).

Male patients comprised significantly more proportion in HBV-related HCC than HCV-related HCC, the serum albumin level was significantly lower in HCV-related HCC, however, the Child-Pugh Class at diagnosis was similar in both groups (Table 1).

### Tumor characteristics and treatment pattern

Large tumor (≥5 cm, 47% vs. 34%, *P*<0.01) and portal vein invasion (27% vs. 16%, *P*<0.01) were more frequent in HBV-related HCC, however, multiple tumor was more common in HCV-related HCC (34% vs. 29%, *P* = 0.03). There was only a marginal difference according to AJCC/mUICC stage between HBV and HCV-related HCC (Table 1). When assessed according to the BCLC staging system (without performance status), the proportion of patients with BCLC stage C or D was higher in HBV-related HCC than in HCV-related HCC (29% vs. 19% and 9% vs. 7%), while the proportion of patients with stage 0, A or B was higher in HCV-related HCC than in HBV-related HCC (9% vs. 7%, 52% vs. 46% and 14% vs. 9%). The proportion of patients diagnosed at within the Milan criteria was significantly lower in HBV- than HCV-related HCC (45% vs. 54%, *P* = 0.01, Table 1). In HBV-related HCC, the proportion of patients diagnosed outside the Milan criteria was highest in age <40 years group (67.4%, 58.0%, 51.3%, 53.3%, and 57.8% for age <40, 40 s, 50 s, 60 s and ≥70 years, respectively).

When stratified according to the treatment modality (curative, palliative vs. none), there was no difference of initial treatment modality between HBV- and HCV-related HCC. Of the specific treatment modality, resection was more common in HBV-related HCC (14% vs. 10%), while local ablation therapy was more common in HCV-related HCC (16% vs. 9%).

### Outcomes

**Figure 2:**
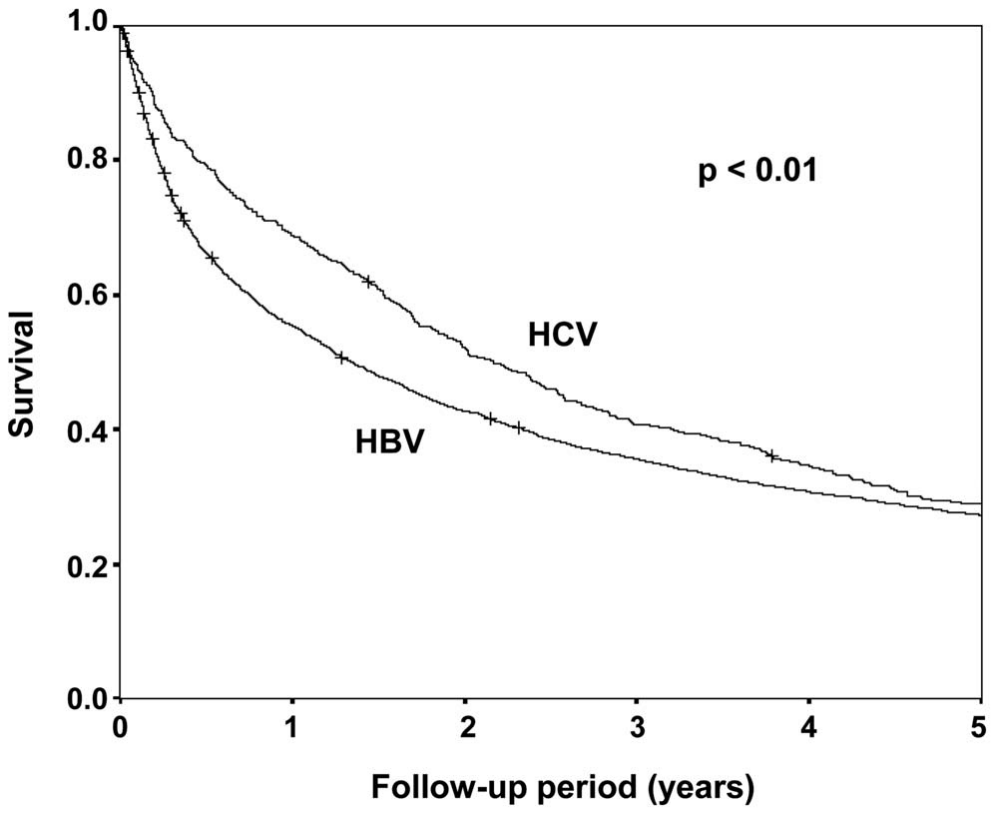
Long-term survival by etiology. The median survival was significantly longer in HCV-related HCC patients than in HBV-related HCC patients. (2.17 vs. 1.34 years, *P*<0.01).

The 1, 3, and 5 year survival rate was 55%, 35%, and 27% for HBV-related HCC and was 68%, 40%, and 28% for HCV-related HCC. The median survival was significantly longer in HCV-related HCC (2.17 vs. 1.34 years, *P*<0.01, Fig. 2). The hazard ratio for survival was significantly lower in HCV-related HCC compared to HBV-related HCC in both un-adjusted and adjusted analysis (Table 2).

**Table 2:** Survival by etiology.

		Model 1		Model 2		Model 3		Model 4	
	Median survival (years)	HR (95% CI)	*P*-value	HR (95% CI)	*P*-value	HR (95% CI)	*P*-value	HR (95% CI)	*P*-value
HBV	1.34	Reference		Reference		Reference		Reference	
HCV	2.17	0.94 (0.84–1.05)	0.28	0.88 (0.78–0.99)	0.04	0.86 (0.77–0.98)	0.02	0.88 (0.78–0.98)	0.03

Abbreviation: HBV, hepatitis B virus; HCV, hepatitis C virus. HR, hazard ratio. Model 1  =  crude hazard ratio, Model 2  =  adjusted for age, gender, Model 3  =  Model 2 + Child-Pugh class and AJCC/mUICC stage, Model 4  =  Model 3 + initial treatment modality.

The independent factor for survival was similar for both HBV- and HCV-related HCC. Gender, Child-Pugh Class, AJCC/mUICC stage and treatment modality was independent factors associated with survival in both HBV- and HCV-related HCC (Table 3). Age (per year) was not an independent factor for survival in HBV-related HCC, while it was an independent factor for survival in HCV-related HCC (Table 3). However, when patients were grouped in 10-year intervals, the median survival was shortest in patients under 40 (0.83 years), followed by 40–49 (1.15 years), over 70 years (1.26 years), 60–69 (1.43 years), and longest in the 50–59 age group (1.58 years) in HBV-related HCC, showing poor survival in both extreme of age group. Patients under 40 showed shorter survival than the age 50–59 group in unadjusted analysis, but when adjusted, patients under 40 showed comparable survival (Table 3). Older age groups (60–69 and ≥70 years) showed significantly shorter survival than 50–59 age group in unadjusted and adjusted analysis. In HCV-related HCC, there were only a few patients in the 40 (*n* = 4) and 40–49 (*n* = 22), so those patients were grouped as under 50 years, 50–59, 60–69 and ≥70 years. Age per year was a significant factor associated with patient survival in HCV-related HCC (Table 3). Older age groups (60s, ≥70 years) showed poorer survival than the 50–59 age group in adjusted analysis (Table 3).

**Table 3:** Comparison of prognostic factors between hepatitis B virus- and hepatitis C virus-related hepatocellular carcinoma.

	HBV-related HCC				HCV-related HCC			
	Unadjusted		Adjusted		Unadjusted		Adjusted	
	HR (95% CI)	*P* value	HR (95% CI)	*P* value	HR (95% CI)	*P* value	HR (95% CI)	*P* value
Age (years)	1.00 (0.99–1.01)	0.16	1.00 (0.99–1.01)	0.13	1.01 (1.00–1.03)	0.01	1.01 (1.01–1.02)	0.03
<40	1.24 (1.04–1.48)	0.01	1.13 (0.95–1.35)	0.15	1.08 (0.64–1.81)	0.77	1.32 (0.78–2.23)	0.29
40–49	1.08 (0.97–1.21)	0.13	0.95 (0.85–1.06)	0.40				
50–59	Reference		Reference		Reference		Reference	
60–69	1.21 (1.08–1.35)	0.01	1.15 (1.03–1.29)	0.01	1.17 (0.87–1.59)	0.28	1.41 (1.04–1.91)	0.03
≥70	1.48 (1.21–1.78)	<0.01	1.22 (1.01–1.49)	0.04	1.53 (1.12–2.08)	0.01	1.46 (1.07–2.00)	0.01
Gender								
Female	Reference		Reference		Reference		Reference	
Male	1.27 (1.13–1.41)	<0.01	1.37 (1.22–1.54)	<0.01	1.06 (0.83–1.35)	0.63	1.37 (1.06–1.78)	0.01
Child-Pugh Class								
A	Reference		Reference		Reference		Reference	
B	2.20 (2.00–2.42)	<0.01	1.64 (1.48–1.81)	<0.01	2.03 (1.61–2.56)	<0.01	1.73 (1.36–2.20)	<0.01
C	2.82 (2.44–3.26)	<0.01	2.54 (2.19–2.95)	<0.01	2.89 (1.92–4.35)	<0.01	3.23 (2.08–5.00)	<0.01
AJCC/mUICC stage								
I	Reference		Reference		Reference		Reference	
II	1.68 (1.37–1.94)	<0.01	1.72 (1.45–2.05)	<0.01	1.43 (0.99–2.05)	0.05	1.16 (0.80–1.67)	0.42
III	3.32 (2.78–3.96)	<0.01	2.76 (2.31–3.33)	<0.01	2.29 (1.57–3.35)	<0.01	1.67 (1.13–2.45)	0.01
IV	6.07 (5.50–7.30)	<0.01	4.75 (3.94–5.72)	<0.01	4.16 (2.74–6.31)	<0.01	2.38 (1.54–3.68)	<0.01
Treatment								
Curative	Reference		Reference		Reference		Reference	
Palliative	2.83 (2.46–3.25)	<0.01	2.44 (2.11–2.81)	<0.01	2.63 (1.93–3.58)	<0.01	2.83 (2.04–3.91)	<0.01
None	7.78 (6.68–9.07)	<0.01	4.97 (4.24–5.83)	<0.01	5.36 (3.81–7.54)	<0.01	4.68 (3.26–6.71)	<0.01

Abbreviation: HBV, hepatitis B virus; HCC, hepatocellular carcinoma; HCV, hepatitis C virus; HR, hazard ratio.

When stratified by tumor burden (AJCC/mUICC stage), survival was not different in AJCC/mUICC I, II, III tumors (Table 4). However, in AJCC/mUICC stage IV, long-term survival was significantly worse in HBV-related HCC than HCV-related HCC. When stratified by treatment, patients who received curative or palliative therapy, there was no significant difference in survival. However, survival was significantly worse for HBV-related HCC for patients who had not received therapy (Table 4). Even in AJCC/mUICC stage IV tumor, there was no significant different in survival for patients who received treatment, and the survival difference was significant for patients who had no therapy (Table 4).

**Table 4:** Adjusted difference in the survival between hepatitis B virus and hepatitis C virus related hepatocellular carcinoma by subgroup.

	Number of patients (HBV vs. HCV)	Median survival, year, (HBV vs. HCV)	Adjusted hazard ratio (95% CI)	*P*-value
AJCC/mUICC stage I	294 vs. 54	6.60 vs. 5.09	1.10 (0.73–1.66)	0.63
AJCC/mUICC stage II	1,172 vs. 206	2.95 vs. 3.19	0.86 (0.71–1.03)	0.10
AJCC/mUICC stage III	776 vs. 120	0.65 vs. 1.71	0.95 (0.76–1.19)	0.70
AJCC/mUICC stage IV	544 vs. 67	0.28 vs. 0.49	0.65 (0.49–0.88)	0.01
Stage IV and received treatment	311 vs. 33	0.48 vs. 0.95	0.69 (0.44–1.08)	0.11
Stage IV and received no treatment	233 vs. 34	0.12 vs. 0.24	0.64 (0.42–0.98)	0.04
Curative treatment	516 vs. 92	NR vs. 6.19	1.09 (0.78–1.53)	0.59
Resection	295 vs. 34	NR vs. NR	0.72 (0.39–1.35)	0.31
Ablation	194 vs. 54	7.06 vs. 4.47	1.13 (0.74–1.73)	0.55
Palliative treatment	1,597 vs. 246	1.48 vs. 2.12	0.95 (0.81–1.11)	0.53
TACE	1,439 vs. 231	1.72 vs. 2.23	0.97 (0.83–1.14)	0.76
None	672 vs. 109	0.18 vs. 0.33	0.75 (0.59–0.95)	0.02

Abbreviation: HBV, hepatitis B virus; HCV, hepatitis C virus; CI, confidence interval; NR, not reached. In each adjusted model, hepatitis B was used as reference for hepatitis C and following variables were adjusted: age, gender, Child-Pugh class, AJCC/mUICC stage, and initial treatment modality.

## Discussion

In this study, HCC patients clearly showed different clinical manifestation according to viral etiology (HBV vs. HCV). HBV-related HCC occurred at a younger age than HCV-related HCC. The annual incidence rate of HBV-related HCC peaked in the 50–59 year age group, while the annual incidence rate of HCV-related HCC gradually increased up to the over 70 year group. In HBV-related HCC, tumor was more likely to be single and larger, and accompanied by portal vein invasion. Overall, the long-term outcome was worse in HBV-related HCC than HCV-related HCC, however, the survival difference existed only for patients with advanced tumor (AJCC/mUICC stage IV). The outcome of each treatment modality (resection, ablation, and TACE) was also comparable between HBV- and HCV-related HCC.

In this study, we could estimate annual incidence rate of HCC by age groups and etiology for the first time in Korea, as the data was based on a nationwide population-based cohort, and could notice significant differences in the incidence rates of HCC by age group and viral etiology. The highest age-specific incidence rates were observed in the 50–59 age group in HBV-related HCC, while the annual incidence rates of HCV-related HCC kept gradually increasing until the ≥70 group. This phenomenon may be explained by the fact that vertical transmission is the major route of HBV acquisition in Korea [24], while HCV is usually acquired in adulthood [19]. HCC rarely developed before the age of 50 years in HCV-related HCC, likewise in countries where HBV is not endemic [13]. However, in HBV-related HCC, the age-specific incidence rate was not low in patient age less than 50 years. A clear gender difference in the age-specific incidence rate of HBV-related HCC was also observed. In men, highest age-specific incidence rates were observed in the 50–59 age group (78.6 per 100,000), while it was highest in the 60–69 age group (20.4 per 100,000) in women. In HCV-related HCC, there was no significant difference in the peak age group between men and women.

Notably, there were significant differences in the tumor characteristic at diagnosis by viral etiology. HBV-related HCC were more likely to be single, large, and accompanied by portal vein invasion. Different oncogenic mechanism by different viruses may explain the observed differences in tumor characteristics. However, it might also be attributable to the currently recommended surveillance policy for HBV and HCV infected individuals in Korea. An HCC surveillance program has been included in National Cancer Screening Program in Korea since 2003 [9]. Patients over 40 year old with risk factors such as hepatitis B, hepatitis C and cirrhosis are recommended to undergo HCC surveillance by ultrasonography and alpha-fetoprotein levels at 6-month interval. Thus, early participation of HCV-infected patients in regular surveillance program before reaching the peak age of HCC development might have a positive influence on the characteristics of tumor at the time of diagnosis. HBV-related HCC patients were diagnosed at younger age, and the proportion of patients diagnosed outside the Milan criteria was also higher in young age group in HBV-related HCC patients. Several studies reported that most of HCC patients diagnosed at young age did not receive regular HCC surveillance, and presented at advanced stage [25], [26]. As this study did not collect information regarding HCC surveillance before HCC diagnosis, careful interpretation is needed. Nevertheless, this data strongly suggest different surveillance strategy (e.g., different age cutoff for starting surveillance) according to the viral etiology is needed.

The decision to undergo HCC surveillance is determined by the level of risk for HCC, which is related to HCC incidence in a pre-defined at-risk population. Although we cannot provide the exact incidence rate of HCC according to each age group and etiology, we could roughly estimate the annual incidence rate of HCC based on population and prevalence rates of HBV and HCV infection in each age group. The estimated annual incidence rates of HCC in HBV infected patients were 0.02%, 0.09%, 0.5%, 0.8%, 1.2% and 1.1% for age 20–29, 30–39, 40–49, 50–59, 60–69 and ≥70 years, respectively. The estimated annual incidence rates of HCC in HCV infected patients were 0%, 0.02%, 0.1%, 0.4%, 0.8% and 0.6% for age 20–29, 30–39, 40–49, 50–59, 60–69 and ≥70 years, respectively. Similar annual incidence rates of HCC were observed in HBV infected patients who were about 10 years younger than in HCV infected patients. Thus, HBV-infected patients may require earlier participation in an HCC surveillance program compared to that of HCV infected patients.

In this study, we observed differences of overall survival according to the viral etiology. However, when stratified by tumor stage, patients who were diagnosed at AJCC/mUICC stage I, II and III, showed similar survival in HBV- and HCV-related HCC. In AJCC/mUICC stage IV tumor, survival was significantly worse in HBV-related HCC. These findings are consistent with the study of Cantarini et al., which reported that HBV-related HCC is more aggressive than HCV-related HCC, especially for HCC diagnosed at an advanced stage [27]. Whether or not viral etiology should be considered in treatment decision is also an important issue. Indeed, several previous studies have tried to assess whether viral etiology is a significant factor determining treatment outcome, and found that viral etiology was not an independent factor for determining outcomes of HCC who underwent resection [28], [29], ablation [30], or TACE [31]. In this study, we also found that viral etiology is not an independent factor associated with survival for patients who received therapy. Even in AJCC/mUICC stage IV tumor, survival was not significantly different when patients received treatment (Table 4). Therefore, although viral etiology should be considered in estimation of patient prognosis, it may have little impact on choosing therapeutic modality.

There are some limitations in this study. Information of some important prognostic factors (e.g., performance status, serum alpha-fetoprotein levels, and use of antiviral therapy, etc.) was not collected. The BCLC stage was not collected and could not be properly assessed. There are also some remained questions whether viral etiology would affect the treatment outcome of other types of treatment, such as transplantation or sorafenib. For liver transplantation, HBV-related HCC has been reported to be an independent factor associated with better survival than HCV-related HCC [32]. For sorafenib, the median survival in the Sorafenib HCC Assessment Randomized Protocol Trial (SHARP) trial (10.7 months) was longer than the median survival of same trial performed in the Asia-Pacific Study (6.5 months) [33], [34]. Unfortunately, sorafenib had not been approved in Korea during period of the present study, so there were no patients who used sorafenib. Hence, it was impossible to assess the impact of viral etiology for the prognosis of patients treated with sorafenib. Lastly, this study only assessed first treatment modality. It is known that HCV-related tumors have higher recurrence rate after curative resection than HBV-related HCC [35], which may have affected long-term outcome.

Despite some limitations, this study provides highly reliable information from unselected nationwide cohort, which minimized selection bias, and large sample size with long term follow-up. HBV-related and HCV-related HCC have clear differences in clinical manifestation, requiring different screening strategy according to etiology to optimize HCC surveillance in HBV-endemic area. However, etiology does not affect treatment outcome and long-term survival within the same stage except for far advanced tumor.
