# Correction: Comparison of Clinical Manifestations and Outcomes between Hepatitis B Virus- and Hepatitis C Virus-Related Hepatocellular Carcinoma: Analysis of a Nationwide Cohort

**DOI:** 10.1371/journal.pone.0116652

**Published:** 2015-01-23

**Authors:** The PLOS ONE Staff

There are errors in the author affiliations. The affiliations should appear as shown here: Dong Hyun Sinn^1,2^, Geum-Youn Gwak^1,2^, Juhee Cho^3,4^, Seung Woon Paik^1,2^, Byung Chul Yoo^1,2^

1 The Korean Liver Cancer Study Group, 2 Department of Medicine, Samsung Medical Center, Sungkyunkwan University School of Medicine, Seoul, Korea, 3 Department of Health Science and Technology, Samsung Advanced Institute for Health Science and Technology, Sungkyunkwan University, Seoul, Korea, 4 Department of Health, Behavior and Society and Epidemiology, Johns Hopkins Bloomberg School of Public Health, Baltimore, United States of America.

## References

[1] Sinn DH, Gwak G-Y, Cho J, Paik SW, Yoo BC (2014) Comparison of Clinical Manifestations and Outcomes between Hepatitis B Virus- and Hepatitis C Virus-Related Hepatocellular Carcinoma: Analysis of a Nationwide Cohort. PLoS ONE 9(11): e112184 doi: 10.1371/journal.pone.0112184 25372403 10.1371/journal.pone.0112184 PMC4221592

